# Successful pregnancy with stage IB2 uterine cervical cancer: A case report

**DOI:** 10.1002/cnr2.1542

**Published:** 2021-09-15

**Authors:** Saya Nagasawa, Makiko Kasahara, Yuji Aoki, Soshi Kusunoki, Yayoi Sugimori, Shozo Matsuoka, Kanako Ogura, Daiki Ogishima

**Affiliations:** ^1^ Department of Obstetrics and Gynecology Juntendo University Nerima Hospital Nerima‐ku Tokyo Japan; ^2^ Department of Diagnostic Pathology Juntendo University Nerima Hospital Nerima‐ku Tokyo Japan

**Keywords:** Apgar score, cervical cancer, conization, lymph node excision, pelvic lymphadenectomy, pregnancy

## Abstract

**Background:**

Although cervical cancer is one of the most common malignancies in pregnancy, its management mainly follows the guidelines for nonpregnant disease state. Within the limited time, patients, and healthcare workers must make difficult decisions to either delay treatment until documented fetal maturity or start immediate treatment based on the disease stage.

**Case:**

The patient was a 37‐year‐old woman: gravida 1, para 0. Her cervical cytology revealed a high‐grade squamous intraepithelial lesion at 8 weeks' gestation. Moreover, invasive squamous cell carcinoma was suspected based on the findings of uterine cervix biopsy. Cervical conization was performed at 11 weeks' gestation, confirming a histopathological diagnosis of squamous cell carcinoma, pT1b2. Cervical cytology findings continued to be negative for intraepithelial lesion or malignancy from 2 weeks after conization until 2 weeks before a cesarean section. In addition, we performed abdominal pelvic lymphadenectomy at 16 weeks' gestation to determine whether the patient could continue her pregnancy. No lymph node metastasis or local recurrence was observed. Finally, a cesarean section and modified radical hysterectomy were performed at 35 weeks' gestation. There was no carcinoma invasion or metastasis. A baby girl weighing 2056 g was delivered with 1‐ and 5‐min Apgar scores of 8 and 9, respectively. Five years postoperatively, there was no evidence of cancer recurrence.

**Conclusion:**

Management of cervical cancer during pregnancy by using a combination strategy of deep conization and pelvic lymphadenectomy could be an effective strategy for carefully and safely assessing risks of recurrence and metastasis.

## 1 INTRODUCTION

Cervical cancer (CC) is one of the most common malignancies in pregnancy, with an estimated incidence of 1.2 to 1.5 cases per 10 000 births.^1^, ^2^ The incidence of abnormal cervical cytologic findings during pregnancy is ~5%–8%.^3^, ^4^ Approximately 1%–3% of women with CC are diagnosed during or after pregnancy.^3^, ^5^ Stage I disease is three times more common in pregnant patients than in non‐pregnant ones, which may be explained by routine prenatal cervical screening.^4^, ^6^ CC during pregnancy is expected to increase due to the marrying‐late trend in Japan. Rarity of the disease and lack of randomized control studies have prevented the establishment of treatment guidelines. Disease management in pregnant women mainly follows the guidelines for nonpregnant disease state, expert opinions, and limited case reports. Invasive CC treatment during pregnancy should be individualized and managed according to cancer stages, patients' willingness to continue their pregnancy, and fetal maturity. Patients must make difficult decisions to either delay treatment until documented fetal maturity or undergo immediate treatment according to their disease stage. Advances in neonatal medicine in recent years have improved the prognosis of premature births and provided varied management methods for these patients. However, there is no established recommendation for the care of pregnant women with CC. The present case report describes a woman diagnosed with CC, 2018 International Federation of Gynecology and Obstetrics (FIGO) stage IB2, during pregnancy in which a cesarean section followed by modified radical hysterectomy was performed at 35 weeks' gestation in our hospital.

## 2 CASE

**FIGURE 1:**
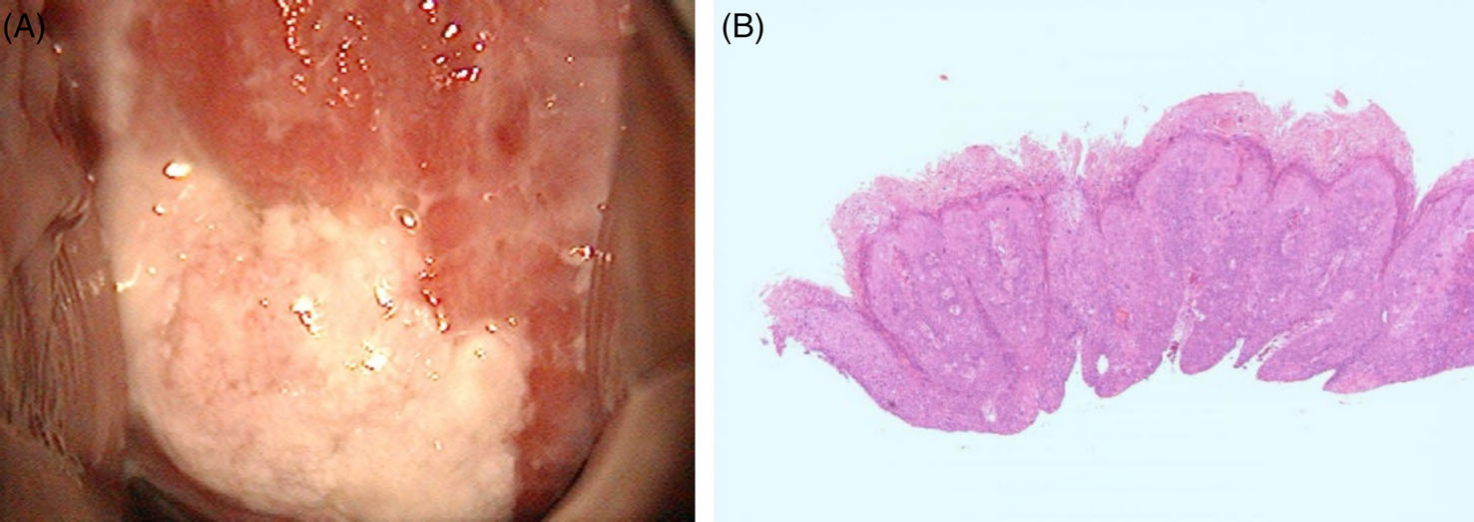
(A) Colposcopy showing a white condyloma‐like lesion on the posterior half of the uterine cervix. (B) Histopathological examination of the cervical biopsy specimen indicates invasive keratinizing‐type squamous cell carcinoma

**FIGURE 2:**
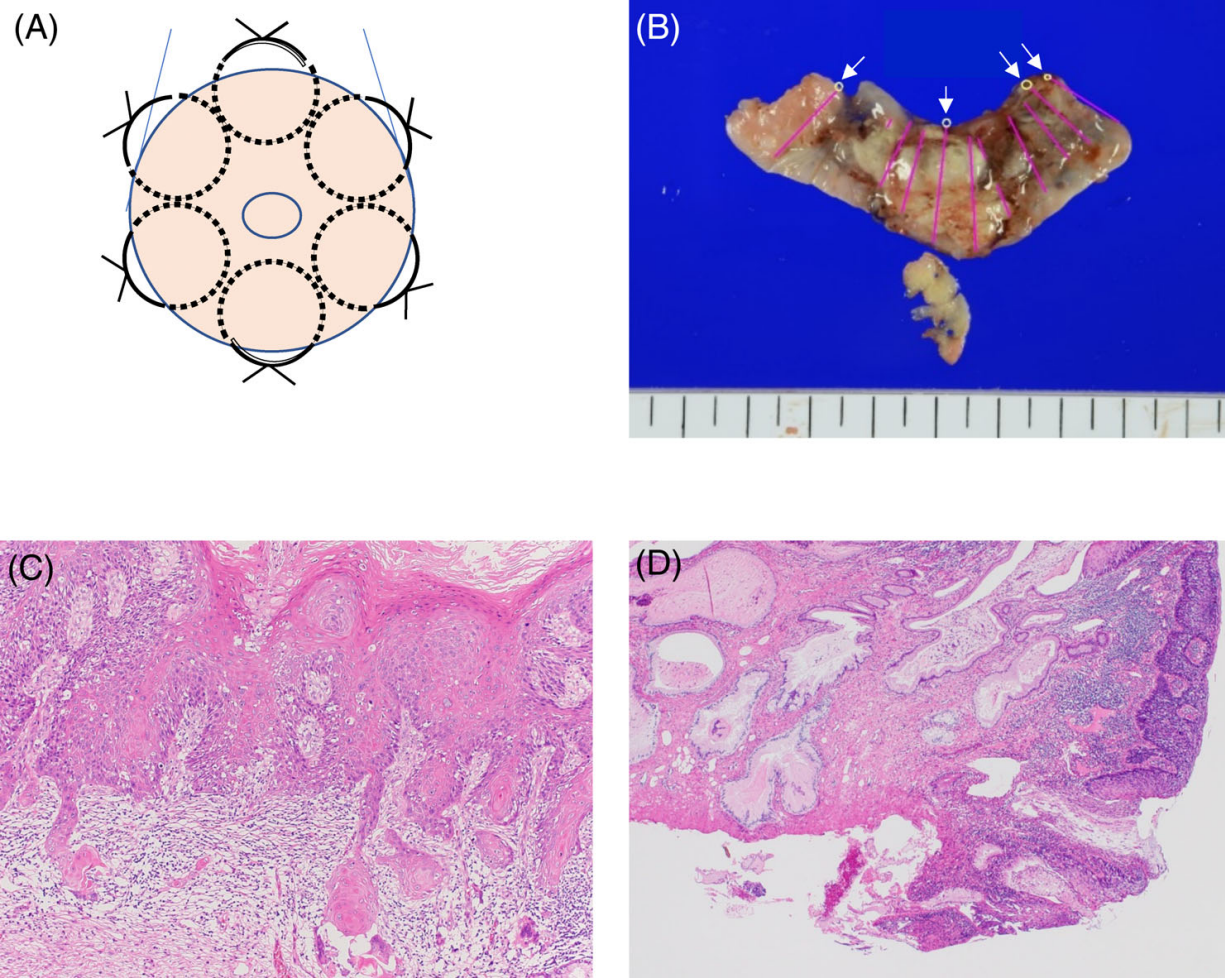
(A) Diagram showing the whole circumference of the uterine cervix was sutured at 2 cm under the vaginal fornix using a 1–0 Maxon suture to decrease blood loss during surgery. (B) Macroscopic findings of cervical conization show that the tumor was removed from almost the entire circumference. White arrows indicate a positive margin area. (C) Microscopic findings of cervical conization show invasive squamous cell carcinoma (hematoxylin and eosin staining, original magnification ×40). (D) The positive surgical margin area is detected by microscopic examination (hematoxylin and eosin, original magnification ×40)

A 37‐year‐old primigravida Japanese woman presented to our hospital in her first trimester with a high‐grade squamous intraepithelial lesion found in a Papanicolaou smear examination during the initial evaluation of her pregnancy. She did not have any relevant family history and had not undergone a cervical smear for at least 15 years. Colposcopy showed a white condyloma‐like lesion in the posterior half of the uterine cervix (Figure 1A). Cervical biopsy at 8 weeks' gestation revealed invasive keratinizing‐type squamous cell carcinoma (Figure 1B). We performed cervical conization at 11 weeks' gestation. At the beginning of this surgery, in order to decrease blood loss, the whole circumference of the uterine cervix was sutured at 2 cm under the vaginal fornix using a 1–0 Maxon suture (Figure 2A). Subsequently, deep conization was performed with only 5 ml of blood loss. The tumor diameter was 2 cm, and the depth of invasion was 1 mm with a positive surgical margin around almost the entire circumference (Figure 2B). Histopathological examination revealed FIGO stage IB2 squamous cell carcinoma without any lymphovascular space invasion (pT1b2, ly0, v0) (Figure 2C,D).

**FIGURE 3:**
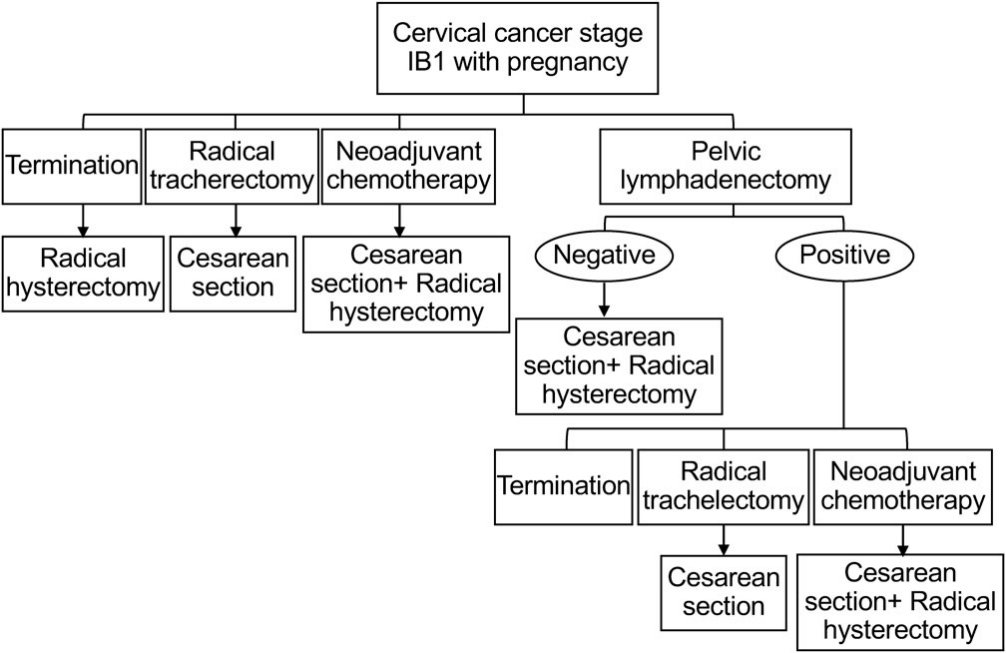
Flow chart of treatment plans of International Federation of Gynecology and Obstetrics stage IB2 cervical cancer with pregnancy

**FIGURE 4:**
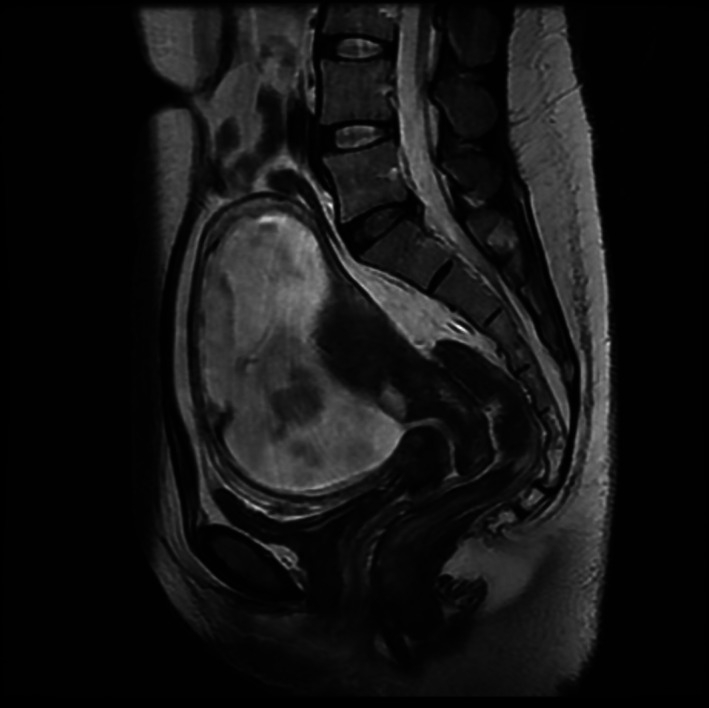
T2‐weighted sagittal magnetic resonance imaging of the pelvic area at 16 weeks' gestation reveals no evidence of remnant cervical cancer

Several treatment plans were suggested as shown in the flow chart in Figure 3. Feasible treatment options were radical trachelectomy, neoadjuvant chemotherapy, or diagnostic pelvic lymphadenectomy. Following in‐depth counseling with relevant specialists with emphasis on potential consequences of deviating from normal practice, the patient and her family chose pelvic lymphadenectomy and decided to continue with the pregnancy for as long as possible. Magnetic resonance imaging was performed at 14 weeks' gestation, revealing no evidence of cervical tumor or distant metastatic disease (Figure 4). Pelvic lymphadenectomy was performed using the laparotomic extraperitoneal approach at 16 weeks' gestation. Intravenous tocolytics were administered after lymphadenectomy for 3 days prophylactically. Histopathological examination showed no evidence of lymph node metastasis (*n* = 0/21). Of note, cervical cytology showed no atypical cells 2 weeks after cervical conization. Therefore, we decided to continue with the pregnancy until the fetus matured sufficiently. Cervical cytology was performed every month, and the results were negative for intraepithelial lesions or malignancy until 2 weeks before the cesarean section. The patient did not have any sign of threatened preterm labor.

An elective cesarean section and modified radical hysterectomy were performed at 35 weeks' gestation. We made an incision in the upper part of the lower uterine segment, not to bother the uterine cervix for the following hysterectomy. A baby girl weighing 2056 g was delivered with 1‐and 5‐min Apgar scores of 8 and 9, respectively, and the baby was discharged from the neonatal unit 2 weeks later without any medical problems. Following delivery, a modified radical hysterectomy was performed using the extraperitoneal approach. Since negative cervical cytology suggested no or slight remnant tumor found in the uterine cervix, we chose modified radical hysterectomy. Without any complication, the duration of surgery was 155 min, the specimen weighed 977 g, and blood loss was 970 ml, including amniotic fluid. No evidence of residual disease was detected on histopathological examination. Thus, no adjuvant therapy was administered. We successfully treated the patient without any adverse events until she could deliver a healthy baby. The patient is still being monitored and has remained disease‐free for 5 years. In addition to the first child described herein, the parents welcomed an adopted baby into their family 5 years after treatment.

## 3 DISCUSSION

Current management of CC in pregnancy is based on recommendations for nonpregnant women. Published reviews indicated that pregnancy itself does not adversely affect the prognosis of CC.^4^, ^7^ Regarding postponement of treatment, the Practice Bulletin by the American College of Obstetricians and Gynecologists stated that delivery can be postponed by 6 weeks.^8^ Unfortunately, in order for the fetus to survive, this recommendation is unsuitable in the Japanese context because cervical screening is performed during the first trimester in Japan.

Colposcopy is recommended for women with suspected uterine cervical dysplasia. Diagnostic cervical conization is indicated even during pregnancy if FIGO stage IA CC is suspected. Treatment of preinvasive disease can be delayed to the postpartum period. The standard surgery for stage IB CC is radical hysterectomy. However, the management of CC during pregnancy depends on the estimated gestational age and willingness of the patient and family. If diagnosed in the third trimester, when the fetal lung sufficiently matures, definitive therapy is recommended for patients with CC. If the patient and her family strongly request to proceed with the pregnancy, there are a few methods of dealing with invasive CC during pregnancy.

### 3.1 Radical trachelectomy

The number of cases of radical trachelectomy during pregnancy has increased. However, complications of this method are serious, such as infertility, constriction of the cervix after surgery, and increasing preterm delivery. Twenty‐two case reports of radical trachelectomy during pregnancy have been published, including 16 live babies (72.7%).^9^, ^10^, ^11^, ^12^, ^13^, ^14^, ^15^, ^16^, ^17^, ^18^, ^19^ Although radical trachelectomy is the most curative treatment during pregnancy, its technical difficulty causes limited access to available institutions. Enomoto et al., reported that from 2011 to 2014, they had four live babies after radical trachelectomy at 15 to 17 weeks' gestation.^16^ Despite their results, previous reports from outside Japan showed that there was little chance to deliver mature babies. Regarding other complications aforementioned, high risks are associated with this surgery.

### 3.2 Neoadjuvant chemotherapy

Some reviews of platinum‐based neoadjuvant chemotherapy have been published recently. During the first trimester, the estimated teratogenic risk for the fetus ranges from 7.5% to 17% with single‐agent therapy, which increases to 25% with combination cytotoxic chemotherapy.^20^ If treatment is provided during the second and third trimesters, the rate of adverse effects is similar to that in normal pregnancies (1%–3%). None of the reported cases showed any fetal abnormality following the use of neoadjuvant chemotherapy during pregnancy. However, the populations were small and self‐selected. Additional outcomes of chemotherapy might include possible intrauterine growth restriction, fetal death, low birth weight, and premature birth. Furthermore, hematopoietic suppression, infertility, retarded development, carcinogenesis, and second‐generation teratogenesis have been observed.^21^, ^22^ Neoadjuvant chemotherapy could delay cancer progression slowly enough to give the fetus enough time to mature. However, it is difficult to cure CC completely with chemical agents only. Dawood et al. reviewed case reports of neoadjuvant chemotherapy from 1996 to 2012.^22^ Among 34 cases of CC in pregnancy, there were 26 cases of stage IB disease and 8 cases of stage II or III disease. Of the 34 cases, complete response was observed in only one case. Partial response was observed in 67.9% of cases, disease stabilization was observed in 21.4% cases; non‐responsive or progressive disease seemed uncommon due to publication bias. At least five patients with stage IB CC died of the disease within 5 years. Neoadjuvant chemotherapy is not the first choice for ensuring the survival of the fetus and mother.

### 3.3 Pelvic lymphadenectomy

For women who wish to continue their pregnancy but are at significant risk for lymph node metastases, staging lymphadenectomy via laparotomic, or laparoscopic approaches may provide important information on the lymph node status. Lymph node involvement is an important prognostic factor for CC.^23^ Several studies have reported the feasibility of lymphadenectomy during pregnancy.^24^, ^25^, ^26^, ^27^ In a study of 31 patients, lymphadenectomy was rarely associated with maternal or fetal morbidity.^14^, ^28^ We chose the laparotomic approach to perform the surgery within a short duration to minimize any possible anesthetic influence on the fetus, paying attention not to press the uterus. Presence of positive nodes modifies therapeutic approaches and alters pregnancy outcomes. Lymph node‐positive pregnant women should terminate their pregnancy or be informed of the need for immediate treatment, including neoadjuvant chemotherapy. Notably, when the tumor diameter is ≤2 cm, the frequency of pathological parametrial invasion and lymph node metastasis is reportedly very low, and relapse‐free survival remains good.^29^, ^30^ Kinney et al., assessed 83 patients with stage IB CC with a tumor volume less than or equal to that of a sphere measuring 2 cm in diameter (4.19 cm^3^) and no tumor in angiolymphatic spaces.^29^ Out of the 83 patients, none had parametrial nodal metastasis. The median follow‐up was 9.8 years, and the Kaplan–Meier estimate of 5‐year disease‐free survival (DFS) was 97.6%. Kristensen et al., also reported that 85 patients with tumors measuring <2 cm or a depth of invasion of ≤10 mm had a 5‐year DFS of 95.3%.^30^ Staging procedures and identification of regional lymph node spread must be performed appropriately.

In the present report, we first performed cervical conization to assess whether the tumor size was ≤2 cm specifically. Following the diagnosis of stage IB2 CC with tumor diameter 2 cm without any lymphovascular space invasion, lymphadenectomy was performed at 16 weeks' gestation revealing no metastasis in the lymph nodes. Both of these results allowed us to observe the patient's pregnancy until 35 weeks' gestation. In addition, there were no atypical cells according to cervical cytology after conization. This is possibly because of local ischemia caused by the tight stitches of the internal ostium. This procedure may delay progression of local tumors. The International Network on Cancer, Infertility and Pregnancy announces guidelines for gynecologic cancers in pregnancy every 5 years. In 2019, they recommended two options for stage IB2 less than 22 weeks' gestation: (i) pelvic lymphadenectomy as a first step followed by either chemotherapy or follow‐up, and (ii) neoadjuvant chemotherapy and subsequent surgical staging of the disease after downstaging the tumor.^31^ Consequently, our strategy was consistent with this new guideline. We additionally recommend deep conization as the first step before lymphadenectomy for CC with tumor diameter ≤ 2 cm.

Time limitation was the most difficult problem for the patient, her family, and the healthcare workers. Cervical conization was performed 3 weeks after her first visit to our hospital. Three weeks later, she had to decide whether to continue her pregnancy or receive the standard treatment for FIGO stage IB2 CC. Fortunately, with her family's support, she made difficult decisions to deliver her baby and save her life. Treatment should be individualized and based on the weeks of gestation, the patient's willingness to continue pregnancy, and the risks of modifying or delaying therapy during pregnancy.

To summarize, this case report describes the management of CC diagnosed during pregnancy with cervical conization and abdominal pelvic lymphadenectomy in the first and second trimesters, respectively. Since the risk of premature birth caused by radical trachelectomy remains high, a combination strategy of cervical conization and pelvic lymphadenectomy could represent an important option for evaluating metastatic risks among pregnant women with CC. Treatment plans are continuously evolving with individualized therapy for optimal outcomes of both the mother and her unborn fetus. Stage at diagnosis, tumor size, nodal status, histological subtype, and gestational age all have influenced the available therapeutic options and outcomes. Each specific case should be discussed and agreed individually when pregnant women are diagnosed with CC. This case indicated that pregnancy could be preserved if the patient had no evidence of lymph node metastasis. The important ethical, emotional, and social development dilemmas of the treatment options for the patient and medical team should also be considered. Therefore, decisions have to be made by the patient and a multidisciplinary team of obstetricians, gynecologists, oncologists, and neonatologists.

## CONFLICT OF INTEREST

The authors declare no conflict of interest.

## AUTHOR CONTRIBUTION

All authors had full access to the data in the study and take responsibility for the integrity of the data and the accuracy of the data analysis. *Conceptualization, supervision*, D.O.; *Management and performance of surgeries* S.K., Y.S., and S.M.; *Performance of histopathological examination, advice*, Y.A. and K.O.; *Writing‐manuscript* S.N. and M.K.

## ETHICAL STATEMENT

The study was performed in accordance with the Declaration of Helsinki.

## INFORMED CONSENT

This patient provided informed consent for the publication of her case and accompanying images.

## Data Availability

Data sharing is not applicable to this article as no new data were created or analyzed in this study.
